# Supplementary material for: Patterns of Intron Gain and Loss in Fungi
Source: PLoS Biol. 2004 Nov 30;2(12):e422. doi: 10.1371/journal.pbio.0020422 (PMC532390; doi:10.1371/journal.pbio.0020422)
Supplement: Table S1 — Also available at http://genes.mit.edu/NielsenEtAl/. (4.3 MB ZIP). [file pbio.0020422.st001.zip › NielsenEtAl/html/555.html]

AN0862.1.NCU03401.1.MG06730.1.FG10822.1


```
 CLUSTAL W (1.82) Multiple Sequence Alignments - Introns Inserted


Sequence 1: NCU03401.1	129 aa
Sequence 2: MG06730.1	135 aa
Sequence 3: FG10822.1	138 aa
Sequence 4: AN0862.1	138 aa
Alignment Length: 142 aa
Number Identitical Residues: 57 aa
Alignment Score (without introns) 2970


MG06730.1 	----MQRRM0LTKEDEVAAG--DEVEVRREDQDKINRFSRLHQRELLLQEELKSKN0KEK
NCU03401.1	----MNRRM0LSKEDEEAAG--DEIEVRREDQDKINRFSRLHQRELALEEELKNKN~KEK
FG10822.1 	MASLVQRRM0LSKADEEAA---DEVEVRREDQDKINRFSRLHQRELVLGDELSAKN0KEK
AN0862.1  	---MMNHRM0LSKEDEAAIGNSEDTEVRREDQEKINRFSRLHQRETVLEEKLKGKQ0KDK
          	    :::** *:* ** * ..::: *******:************  * ::*. *: *:*

MG06730.1 	EELDDITMELELA-DEDESVP~YKVGDAFFHVPLPQAQEMLSTSATKVEGQIEDIEDRLG
NCU03401.1	EELDDLETELELVEDEDELVQ2YKIGDSFFHIPLEQAKEMLGIAAAKLIKEIEALDDKLE
FG10822.1 	EELDDLSTELELA-DEDEKIQ2YKIGDAFFHVSVEQAQEMLEKATETLEEESTSLEDKLS
AN0862.1  	EDLEEISTELELA-DEDELIP2YKIGDSFVHLPLEEAQTLLASSTEQIDSEVAKLEETLS
          	*:*:::  ****. **** :  **:**:*.*:.: :*: :*  ::  :  :   ::: * 

MG06730.1 	SIREEMTQLKVEL~YARFGKSIQLET
NCU03401.1	TIRKEMTGLKVEF2SIDQG-------
FG10822.1 	SIREEMTKLKVDL~YARFGKQINLET
AN0862.1  	DLRDEMQQLKVAL~YARFGRSINLET
          	 :*.**  *** :     * . . .:
```
